# Data analysis for behavioral medicine: an introduction to the special issue

**DOI:** 10.1007/s10865-026-00661-7

**Published:** 2026-05-29

**Authors:** Mauricio Garnier-Villarreal, Alexander Schoemann, Manshu Yang

**Affiliations:** 1 Vrije Universiteit Amsterdam, Amsterdam, Netherlands; 2 East Carolina University, Greenville, NC USA; 3 University of Rhode Island, Kingston, USA

**Keywords:** Quantitative Methods, Research Methods, Mixture Models, Longitudinal Models, Location-Scale Models, Causality, Bayesian

## Abstract

Methods and theory are deeply intertwined in behavioral research: theoretical developments lead to the need for new methods, and methodological innovations, in turn, open the door to new theoretical insights. To continue advancing research in behavior medicine, new methods need to be developed and implemented. At the same time, data collected in this field are becoming increasingly complex, requiring quantitative approaches that are both rigorous and accessible to analyze them effectively. In this Special Issue we have included eighteen papers that highlight developments and applications of quantitative methods for behavioral medicine. Across the papers we identified six main themes: mixture modeling, longitudinal modeling, location-scale models, methods for randomized trials and causal inference, methods using Bayesian inference, and estimating complex, nonlinear relationships.

## Introduction

This special issue contains contributions on the development and dissemination of methods for collecting, analyzing, and interpreting data used in health-related research. Papers in this special issue include methodological research developing new methods (e.g. Garnier-Villarreal 2026; Zhang, 2026), examples of how advanced methods can be implemented in behavioral medicine research (e.g. Bouwhuis, 2026; Dousti Mousavi, 2026; Mara, 2026; Wang, 2026), and tutorials on implementing advanced methods (e.g. Moreno-Villamizar, 2026). We have identified the following themes that may guide future research in behavioral medicine: mixture modeling, longitudinal modeling, location-scale models, methods for randomized trials and causal inference, methods using Bayesian inference, and estimating complex, nonlinear relationships. Many of the papers in this special issue span multiple of these areas, for example Mara et al. (2026) examines longitudinal modeling in randomized trials, Myers et al. (2026) uses longitudinal mixture models in the context of randomized trials, and Bouwhuis et al. (2026) uses mixture models in longitudinal research.

### Mixture modeling

Mixture modeling is a method to identify unobserved groups based on responses to a given set of items. Mixture modeling includes a number of different methods such as latent class analysis, latent profile analysis, latent transition analysis, and hidden Markov Models. In these methods, identifying unobserved groups, examining how group membership changes over time, and examining predictors and outcomes from group membership may be of interest. Moore et al. (2026) provides a tutorial on using latent profile analysis to identify unobserved groups with an example using goal orientation in the context of physical activity. Schmeige et al. (2026) use Growth Mixture Modeling (GMM) and repeated measures latent profile analysis (RMLPA) to describe mechanisms of exercise behavior maintenance; where GMM describes the wave-level behavior, and RMLPA describes the short term behavior changes. Myers et al (2026) use latent transition analysis in the context of a randomized trial of physical activity. The goal of the study is to examine how class membership changes over time differ between randomized conditions. Bouwhuis et al. (2026) use cross-lagged hidden Markov models to identify which combinations of job demands and resources (JDR) occur among workers, how often, and how these affect mental health and vice versa. They identified six JDR states, and three mental health states and evaluated the longitudinal relation between the two categorical latent variables over time.

### Longitudinal modeling

Using longitudinal data to examine change over time has a long history in behavioral medicine and psychology. By measuring individuals on multiple occasions it is possible to examine both intraindividual changes and interindividual differences in change. Methods for modeling longitudinal data have progressed rapidly from within-subjects ANOVAs to modern methods using a wide variety of techniques. More than half of the articles involve methods for longitudinal data, often in conjunction with other methods, e.g. Bouwhuis et al. (2026), Myers et al. (2026), and Schmeige et al. (2026) all investigate mixture models with longitudinal data. Additionally, several papers provide tutorials or develop extensions of traditional longitudinal models that are designed to investigate change over time.

Cho and colleagues (2026) compare traditional methods for longitudinal data, repeated measures ANOVA, to a modern modeling alternative, the two-wave latent change score model (2W-LCSM). The 2W-LCSM is a more flexible model that can address measurement error and capture intraindividual change over time. The 2W-LCSM is illustrated with an example highlighting the flexibility of the 2W-LCSM where changes in risk factors predict later recidivism. Moreno-Villamizar and colleagues (2026) present a tutorial for the application of the Random-Intercept Cross-Lagged Panel Model (RI-CLPM) with a relevant example for behavioral and health research. This paper demonstrates that RI-CLPM can help accurately identify psychological mechanisms and processes during behavioral interventions in health settings, aiding intervention personalization. Garnier-Villarreal and Johnson (2026) develop and present a Bayesian Piecewise longitudinal model, that identifies random change points in cognitive tests before or around the time of Mild Cognitive Impairment Diagnosis in Alzheimer's patients. This model tests sample and subject parameters, providing relevant diagnostic information for each subject.

### Location-scale models

The majority of statistical methods in medical and behavioral sciences focus on differences between means, e.g. t-tests, or ANOVAs, or predicting an individual’s score, e.g., regression models. These models ignore additional information in the data beyond the mean, or expected, score of an outcome. In many situations predicting differences in the variability of scores can provide additional insights for theory. Location scale models allow researchers to predict both the mean score (location), and variability of scores (scale) from other variables. These models are especially popular in longitudinal or nested data, where variances can be predicted at multiple levels of analysis. For example, Whitaker and colleagues (2026) present a tutorial on using mixed-effects location scale (MELS) models for examining intra-individual variability in longitudinal data. The tutorial uses data on chronic pain and fatigue and presents examples of increasingly complex models for examining intra-individual variability.

Wang and colleagues (2026) evaluated the estimation accuracy of intraindividual means and variances in ecological momentary assessment data, by comparing values computed using standard computational formulas (SCF) with those estimated via random effects from a MELS model, and found that the MELS approach consistently outperformed the SCF method. MELS models can also be extended and combined with other methods, Zhang and colleagues (2026) combine MELS modeling with mixture modeling. Their model introduces an innovative approach for analyzing intensive longitudinal behavioral data that exhibit hidden patterns in both mean (location) and intraindividual variability (scale) trajectories. The method uses location–scale regressions with latent classes specified in both location and scale components of the model. Its performance is evaluated through a simulation study and illustrated with an application to calorie‑intake data from a weight‑loss management study.

## Methods for randomized trials and causal inference

Understanding if one variable causes changes in another is fundamental for research across behavioral and health sciences. The gold standard for assessing causation in these areas has been the randomized trial where individuals are typically randomly assigned to a treatment or control group and differences between these groups provide an estimate of the causal effect of treatment. Modern randomized trials rarely use a simple randomization scheme with a single measurement after treatment. One popular design is a pretest-postest-follow-up design where participants are randomized then measured before treatment, immediately following treatment and at a later follow-up time. Mara (2026): presents Latent Change Models (LCMs) as a practical approach for analyzing randomized pretest–posttest–follow-up (RPPF) trials, directly estimating discrete changes between timepoints and intervention–control group differences. An important factor in planning randomized studies is determining sample size. The majority of methods for power and sample size determination are based on two-tailed tests, whereas many randomized trials are designed to test one-tailed hypotheses. Liang and colleagues (2026) present a method to convert the power based on a two-tailed test into power for a one-tailed test. They provide both R code and Excel calculators that implement this method.

In other situations randomization is not feasible and alternative methods are required to examine causal effects. In a multigroup cohort study, two groups are compared, based on exposure to a condition that is not randomly assigned. To accurately compare the exposed and unexposed group, the two groups should be as similar as possible outside of exposure. Montoya and colleagues (2026) introduce a method for matching participants, via propensity score matching, during sequential recruitment. The method is illustrated and compared to existing methods using data on PTSD and cardiovascular risk.

Mediation models assume causal relationships between the predictor, mediator, and outcome variable but the majority of mediation models are based on cross-sectional data using methods where it is not possible to establish causality. Loeys and colleagues (2026) address, this issue by introducing an interventional effects method. The interventional effects method provides clear causal reasoning for effects in a more flexible framework than other methods for causal inference in mediation models, e.g. natural direct and indirect effects. They illustrate the interventional effects method in a model where recreational physical activity and dietary behavior mediate the relationship between family affluence and mental well-being.

### Bayesian methods

Methods using Bayesian inference and Markov Chain Monte Carlo (MCMC) methods have grown dramatically in behavioral and health research. These methods allow for more flexibility in inferences and model specification than traditional methods such as null hypothesis testing and maximum likelihood estimation. Several papers in this special issue leverage the combination of Bayesian inference and MCMC to advance methods for behavioral medicine. As discussed in the longitudinal modeling section, Garnier-Villarreal and Johnson (2026) use Bayesian Piecewise longitudinal models to examine cognitive decline. Munion and colleagues (2026) use Bayesian Model Averaging (BMA) to examine networks of symptoms of general and disease specific anxiety in a sample of cardiovascular patients. Models using BMA provide a method to determine the optimal network model identifying links between general anxiety measures and cardiac anxiety. Li and colleagues (2026) use Bayesian nonparametric (BNP) clustering to examine clusters of co-occurring symptoms in cancer survivors. BNP uses machine learning to identify unique subgroups within a dataset. Li and colleagues identify three subgroups of symptoms using BNP and then examine if different treatment types have differential effectiveness in symptom reduction in these subgroups.

## Complex non-linear relationships

Many quantitative methods used in behavioral medicine assume linear relationships between variables. Linear relationships between variables may be a reasonable simplification in some cases but often there are more complex non-linear relationships between predictors and outcomes. Dousti Mousavi and colleagues (2026) examine methods for modeling count data, especially in studies on substance use. They demonstrates the superior performance of the zero-inflated beta-binomial (ZIBB) and beta-binomial hurd (BBH) models in analyzing count data from substance use studies, revealing the effectiveness of the ZIBB model in capturing the U-shaped distribution observed in real-world data. Stadtbaeumer and colleagues (2026) examine linear, e.g. ridge regression, and non-linear machine learning, e.g. random forest, methods for predicting cancer fatigue. A simulation study examines differences between the methods, and the authors discuss using simulation studies as a tool for selecting optimal modeling methods.

## Conclusions

Across the papers in this special issue we identified six main themes: mixture modeling, longitudinal modeling, location-scale models, methods for randomized trials and causal inference, methods using Bayesian inference, and estimating complex, nonlinear relationships. However, it should be clear that these are not six unrelated areas of quantitative methods, rather these different methods (and many other methods) are often combined to answer novel and important questions. Moving forward we expect that this trend will continue, methods will be combined across many different areas to extend the types of hypotheses that researchers in behavioral sciences can examine.
